# Optimizing energy consumption in radiotherapy: standard vs. hypo-/ultra-hypofractionation and becoming SMART (specific, measurable, achievable in radiotherapy)

**DOI:** 10.1007/s00066-025-02419-7

**Published:** 2025-06-17

**Authors:** Ann-Katrin Exeli, Andreas Lurtz, Linda Agolli, Daniel Habermehl

**Affiliations:** Department of Radiotherapy, Giessen, University Hospital Giessen-Marburg, Klinikstr. 33, 35392 Giessen, Germany

**Keywords:** Radiotherapy, Hypofractionation, Ultra-hypofractionation, Sustainability, Carbon footprint

## Abstract

**Purpose:**

The healthcare sector is a large greenhouse gas producer. Especially in radiotherapy (RT), a lot of electricity is consumed by the medical linear accelerator (linac) and associated patient travel. Our aim was to ascertain by how much electrical energy consumption and patient travel can be reduced by replacing normofractionated (NF) with emerging moderately hypofractionated (UF) or ultra-hypofractionated (UHF) concepts.

**Methods:**

We connected an energy meter to our linac (VersaHD, Elekta©, Stockholm, Sweden) and evaluated different fractionation concepts (NF, HF, UHF) for 30 patients with target volumes of the prostate, breast, and spine. In addition to the energy measurements, we also conducted an analysis of the carbon dioxide (CO_2_) emissions associated with the variations in patient travel.

**Results:**

This study measured the energy consumption of a linac (in kWh) and its impact on CO_2_ emissions for various radiotherapy fractionation concepts. Ultra-hypofractionated regimens consistently showed the lowest energy consumption and variability across prostate, breast, and bone metastasis treatment courses, while NF regimens had significantly higher energy consumption and variability. Transitioning from NF to UHF regimens reduced CO_2_ emissions by up to 75%, driven by fewer patient visits and lower electricity consumption. These findings highlight the environmental and logistical benefits of HF and UHF treatment protocols.

**Conclusion:**

The adoption of HF and UHF treatment concepts can significantly reduce energy consumption and CO_2_ emissions, achieving an up to 75% reduction per treatment course. This is primarily due to decreased patient travel and electricity consumption at the linac. Extrapolated globally, these changes offer further potential to mitigate climate change.

## Introduction

The climate crisis is one of the central issues of our time, as it is accompanied by fundamental changes to living conditions on our planet and is leading to a global health crisis [1]. It therefore represents one of the biggest challenges for the healthcare sector worldwide, which itself is responsible for a large proportion of greenhouse gas emissions [2, 3]. The healthcare sector is responsible for around 5.2% of total CO_2_ (carbon dioxide) emissions in Germany. Globally, the healthcare sector is responsible for 4.4% of emissions (2 gigatons of CO_2_/year). For this reason, the World Health Organization (WHO) demands that countries reduce their greenhouse gas emissions in the health sector and improve environmental sustainability [4]. National and international professional societies for radiotherapy (RT), such as the DGMP (Deutsche Gesellschaft für Medizinische Physik), DEGRO (Deutsche Gesellschaft für Radioonkologie), or ESTRO (European Society for Radiotherapy and Oncology), have dedicated themselves to the topic of sustainability and climate protection with newly founded working groups. Manufacturers of linear accelerators are also paying increasing attention to this topic with words such as “sustainability” in their agenda [5].

Radiation oncology is a central component of modern cancer therapy and requires use of energy-intensive technologies and high patient logistics, which entail considerable energy consumption [6]. The potential environmental impact of RT and the associated secondary effects on human health were recently impressively demonstrated in a comprehensive analysis [7]. This highlights the imperative to enhance energy efficiency in this area while safeguarding the high qualitative standards of patient care. The saving potential would be particularly important in RT, where energy-intensive large-scale equipment such as medical linear accelerators (linacs) and various imaging technologies are used on a daily basis. Furthermore, the necessity of logistical effort is frequently evident due to the duration of the treatment, which may extend over several weeks. Hypo- and ultra-hypofractionated concepts have become the new standard for a variety of RT indications in recent years [8–10]. These regimens enable a shorter treatment time with equal clinical outcome and are at the same time resource saving in terms of personnel, carbon dioxide emissions, costs, and energy consumption [11]. The data on mean consumption during a working day for a medical linac in the literature vary greatly in the range of 65–112 kilowatt-hours (kWh), depending on the linear accelerator manufacturer, model, and used mode [12].

The objective of this study is to analyze the energy consumption of our linac. For this purpose, patient groups were selected that represent the most frequent RT indications in our department: prostate, breast, and bone metastases [13–16]. Treatment plans with varying fraction doses were devised for ultra-hypofractionation (UHF), moderate hypofractionation (HF), or normofractionation (NF). The respective fraction dose was based on the fractionation concepts commonly used in our department. The radiation plans were irradiated, and the energy consumption in kWh per treatment plan was measured using an energy meter. In order to present the overall picture of the CO_2_ emissions of the patient groups under consideration and the influence of different RT protocols, we also calculated the patient journeys. Furthermore, the reduced electricity consumption was converted into a carbon dioxide equivalent (CO_2e_) according to the CO_2_ converter of the German Federal Environment Agency as a benchmark for sustainability [17].

## Methods

### Measurement of power consumption

**Fig. 1:**
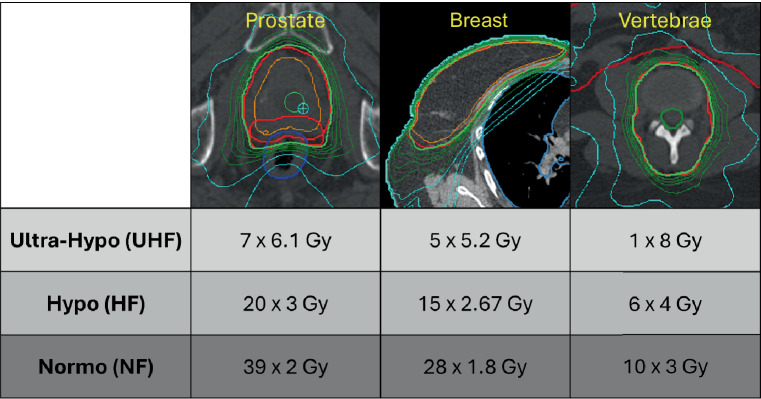
Fractionation schemes for the collective divided into ultra-hypofractionated (*UHF*), hypofractionated (*HF*), and normofractionated (*NF*) regimens for the investigated entities of prostate, breast, and vertebrae. An exemplary dose distribution is shown for each entity, with the green isodose corresponding to 95% of the prescribed dose

We connected a Shelly 3EM (Shelly, Sofia, Bulgaria) to the power cabinet of a VersaHD (Elekta©, Stockholm, Sweden) linac. The Shelly 3EM is a smart electricity meter that can be used to monitor consumption in real time [18]. Dashboards can be used to display voltage in volts, current in amps, power in watts, and energy consumption in kWh (kilowatt-hour). The investigated group comprised a total of 30 patients, who were equally divided into the irradiated entities of prostate, breast, and spine. The treatment planning system was Pinnacle^3^® (version 16.2.1; Philips Healthcare, Amsterdam, the Netherlands). Prostate and spine patients were irradiated with volumetric modulated radiation therapy (VMAT) treatment plans (one and two rotations, respectively) with 6‑MV (megavolt) photons. For breast cancer patients, hybrid plans were calculated (consisting of static and rotating fields) in addition to VMAT-only RT plans. Three fractionation schemes were examined for each entity according to Fig. 1. Depending on the number of fractions, these schemes are referred to as NF, HF, and UHF, with NF having the highest and UHF the lowest number of fractions. The energy consumption per treatment is shown in the results as a boxplot diagram which includes the interquartile range (IQR) as the range between the 25th and the 75th percentile. Whiskers cover the data within 1.5 × IQR. Anything beyond the whiskers is considered an outlier. The energy consumption in the boxplots is normalized to the average energy consumption using UHF protocols, which simplifies readability by focusing on relative ratios, as absolute kWh values can vary significantly between linear accelerator models and manufacturers.

The fractionation schemes used for RT of the prostate are analogous to those of the Hypo-RT prostate cancer trial for UHF fractionation [19], which was the UHF concept with the highest evidence at the time we performed our analysis. Furthermore, this concept can be applied with standard linacs without additional stereotactic equipment or intrafractional image guidance. For the HF plans, we calculated treatment plans with a moderate fractionation scheme according to the PROFIT trial with a total dose of 60 Gy (20 fractions) [20]. For breast RT we calculated plans as a UHF scheme using a five-fraction schedule corresponding to Brunt et al. published in the *Lancet *in 2020 [21]. As the HF concept we applied 15 fractions to 40.05 Gy according to the START‑B trial [22]. A total of 28 fractions with a fraction dose of 1.8 Gy to total doses of 50.4 Gy represented the NF concept, which still has a recommendation in the German S3 breast cancer guideline [23]. Treatment plans for spinal RT cases were dosimetrically divided into a UHF concept with a single fraction of 8 Gy and six fractions to 24 Gy in HF; NF was applied in ten fractions, each with 3 Gy. Fractionation schemes are derived from the current literature and existing guidelines [10].

### Calculations of patient logistics

Changes in the fractionation schedules have direct implications for patient logistics, as travel distance and visit frequency impact CO_2_ emissions. We assumed the number of hospital visits to be the number of fractions plus one visit per patient for initial consultation, patient education, and planning computed tomography (CT). As a reference for the distance, we used a mean distance of 57.7 km, which was calculated from a survey of our patients regarding their travel distances on an exemplary clinical treatment day. This resulted in a total amount of 9.7 kg of CO_2_ emissions for each visit. As an emission factor for the conversion of electricity into CO_2e_, we assumed a value of 0.38 $\frac{\mathrm{kg}\mathrm{C}\mathrm{O}_{2}}{\mathrm{kWh}}$, which corresponds to the German electricity mix in 2023 [24].

## Results

### Measurement of power consumption

First, we analyzed the average energy consumption of our medical linear accelerator per working day. The energy consumption on working days is, on average, approximately 50 kWh over a 12-hour period. On non-working days, the linac was in sleep mode and consumed 5 kWh per day (24 h) on average. This results in a total energy consumption of 52.5 kWh per working day for patient treatment due to the additional 2.5 kWh for the remaining 12 h in the sleep mode of the linac. The energy consumption of our linear accelerator is substantially reduced in sleep mode (approximately 0.2 kWh). As soon as the linac was switched on, the energy consumption rose sharply without the beam being on (3–4 kWh). As soon as the beam was switched on, we detected peaks of up to 20 kWh, while these peaks were only very short lived. On average, the hourly energy consumption observed in this work was nearly 5 kWh in clinical applications.

**Fig. 2:**
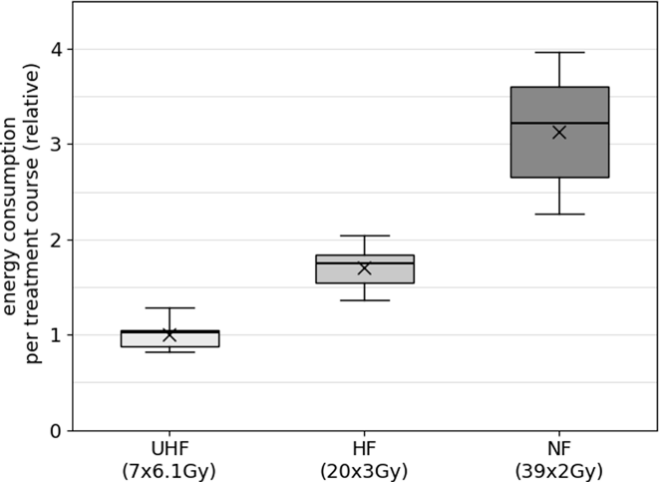
Energy consumption (kWh) per treatment course for prostate cases analyzed for ultra-hypofractionation (*UHF*; 7 fractions at 6.1 Gy/fraction), hypofractionation (*HF*; 20 fractions at 3 Gy/fraction), and normofractionation (*NF*; 39 fractions at 2 Gy/fraction). The respective energy consumption is shown relative to the mean energy consumption for the UHF VMAT treatment plans; “x” marks the mean value of each parameter, the line the median; the box represents the middle 50% of the data (interquartile range, IQR), while the whiskers include the data within 1.5 × IQR

**Fig. 3:**
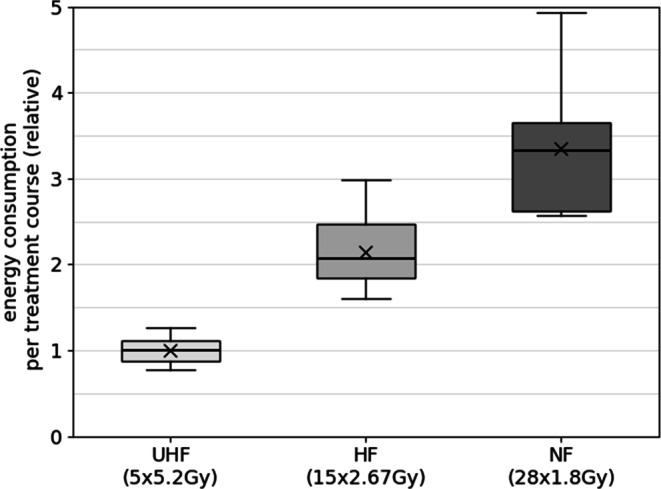
Energy consumption (kWh) per treatment course for the breast cases analyzed for ultra-hypofractionation (*UHF*; 5 fractions at 5.2 Gy/fraction), hypofractionation (*HF*; 15 fractions at 2.67 Gy/fraction), and normofractionation (*NF*; 28 fractions at 1.8 Gy/fraction). The respective energy consumption is shown relative to the mean energy consumption for the UHF treatment plans; “x” marks the mean value of each parameter, the line the median; the box highlights the middle 50% of the dataset (interquartile range, IQR), whereas the whiskers span datapoints within 1.5 × IQR

**Fig. 4:**
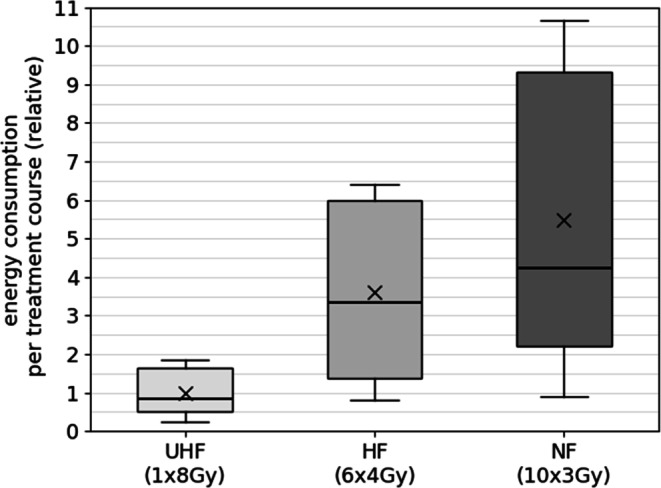
Energy consumption (kWh) per treatment course for ten vertebral bodies cases analyzed for ultra-hypofractionation (*UHF*; 1 fraction at 8 Gy/fraction), hypofractionation (*HF*; 6 fractions at 4 Gy/fraction), and normofractionation (*NF*; 10 fractions at 3 Gy/fraction). The respective energy consumption is shown relative to the mean energy consumption for the UHF VMAT treatment plans; “x” marks the mean value of each parameter, the line the median; the box indicates the middle 50% of values (interquartile range, IQR), with whiskers extending to datapoints within 1.5 × IQR

The boxplots in Figs. 2, 3 and 4 demonstrate the comparative energy consumption in kWh per treatment course for the respective entities: prostate (Fig. 2), breast (Fig. 3), and vertebrae (Fig. 4). Additionally, the mean monitor units (MU) for the treatment plans per entity and concept are summarized in Table 1. The energy consumption shown in the following figures is normalized to the respective averaged energy consumption while using the UHF protocols.

**Table 1:** Mean monitor units (*MU*) and range for the plans per entity (prostate, breast, spine) and fractionation concept

Fractionation concept	Prostate	Breast	Vertebrae
UHF, mean MU (range)	2230.5 (1761.1–2952.5)	1582.6 (1185.6–2132.4)	2041.8 (1613.8–2856.5)
HF, mean MU (range)	1097.0 (866.1–1452.0)	812.6 (608.8–1094.9)	1020.9 (806.9–1428.3)
NF, mean MU (range)	731.3 (577.4–968.0)	547.8 (410.4–738.1)	765.7 (605.2–1071.2)

*NF* normofractionation, *HF* hypofractionation, *UHF* ultra-hypofractionation

The UHF concept in the prostate group shows the lowest median energy consumption, with minimal variation (Fig. 2). The IQR is narrow, and the whiskers indicate a small spread, suggesting consistent energy usage across treatments. The HF concept (20 × 3 Gy) consumes slightly more energy than the UHF concept. The median is around 1.7, and the IQR is slightly wider, indicating greater variability in energy consumption compared to UHF concepts. The NF concept (39 × 2 Gy) has the highest energy consumption, with a median value of just over 3 per treatment course. The boxplot also shows a wider IQR compared to the other two regimens, indicating greater variability in energy usage. The whiskers extend further, suggesting that this treatment plan involves more variability in energy consumption across different treatment sessions.

Figure 3 shows the breast collective, in which the UHF concept demonstrates the lowest median energy consumption. The IQR is slim, suggesting low variability in energy use. The HF concept shown in the middle exhibits a moderate increase in energy consumption compared to the UHF regimen, with an approximately doubled median energy consumption. The NF regimen has the highest median energy consumption, around 3.3 per treatment course. The IQR is larger than in the other two regimens, indicating more variability in energy use. The whiskers are noticeably longer, suggesting considerable fluctuation in the energy consumption across treatments. Overall, the boxplot for the breast collective highlights a clear trend of increasing energy consumption per treatment course from the UHF to the NF concept. Additionally, the variability in energy consumption increases progressively from the UHF regimen to the NF regimen, with shorter treatment courses (UHF) being associated with lower and more consistent energy usage, while longer courses (NF) consume more energy and exhibit greater variability.

The results for the bone metastasis protocol are shown in Fig. 4. The UHF regimen shows the lowest energy consumption per treatment course. The HF group has a median energy consumption near 3.3, considerably higher than the UHF regimen. The NF regimen has the highest energy consumption, with a median value around 4.2 and, on average, 5.5 times higher energy consumption per treatment course. The IQR is large, signaling variability in energy use across different treatment courses. The whiskers extend much further than in the other two regimens, illustrating considerable spread and variability in energy consumption. The charts highlight a clear pattern of increasing energy consumption per treatment course from the UHF to the NF concept.

Based on our measurements, the absolute energy required for a course of therapy was approximately 174 kWh (NF), 106 kWh (HF), and 53 kWh (UHF) for breast RT and 195 kWh (NF), 120 kWh (HF), and 71 kWh (UHF) for prostate RT. For vertebral body RT, the absolute energy consumption per treatment course was 55 kWh (NF), 36 kWh (HF), and 11 kWh (UHF).

### Influence of the change in fractionation on CO_2_ emissions due to patient visits

The CO_2_ emissions can be categorized into two distinct components: those attributable to patient travel and those associated with the energy consumption of the linac (Table 2). The latter CO_2e_ are calculated using the energy consumption at the linac and an emission factor of 0.38 $\frac{\mathrm{kg}\mathrm{C}\mathrm{O}_{2}}{\mathrm{kWh}}$ [24]. Patient visits were obviously reduced due to the reduction in fractionation, and, thus, fewer CO_2_ emissions were generated using UHF concepts. In the breast cancer collective, the number of total visits decreased from 30 (NF) to 17 (HF) and 7 (UHF), resulting in CO_2_ emissions of 356.9 kg, 205.1 kg, and 88.0 kg, respectively. This shows a percentage reduction of 43% from NF to HF and of 75% from NF to UHF. For the group of prostate cancer patients, a reduction in the number of visits from 41 (NF) to 22 (HF) and 9 (UHF) resulted in CO_2_ emissions of 471.5 kg, 258.9 kg, and 114.2 kg, respectively, representing relative reductions of 76% (NF to UHF) and 45% (NF to HF). In the patients with bone metastases, a reduction in the number of visits from 12 to 8 and 3 reduced the emissions from 137.2 kg to 91.2 kg and 33.3 kg. This resulted in a percentage reduction of 34% from the normofractionated concept to the more significant hypofractionation with 6 fractions and of 76% for the single-fraction concept. These calculations are based on the idealized assumption that all patients come by car or cab. A patient survey revealed that of 53 patients, 47 came to treatment by car or cab, which corresponds to 89%. Each patient travelled, on average, 57.7 km (range 2.6–83.3 km, median 28.1 km) to and from the hospital each day.

**Table 2:** CO_2_ emissions (kg) according to fractionation protocol including patient travel and linac power consumption (CO_2e_ in kg)

Patient group	Protocol	Total number of visits	CO_2_ emissions due to visits (kg)	Power consumption linac (kWh)	CO_2_ emissions by linac (kg)^a^	CO_2_ emissions due to visit and linac (kg)
Breast	NF	30	290.8	174	66.1	*356.9*
	HF	17	164.8	106	40.3	*205.1*
	UHF	7	67.9	53	20.1	*88.0*
Prostate	NF	41	397.4	195	74.1	*471.5*
	HF	22	213.3	120	45.6	*258.9*
	UHF	9	87.2	71	27.0	*114.2*
Vertebrae	NF	12	116.3	55	20.9	*137.2*
	HF	8	77.5	36	13.7	*91.2*
	UHF	3	29.1	11	4.2	*33.3*

Total number of visits includes fraction number + first outpatient contact + CT planning

*NF* normofractionation, *HF* hypofractionation, *UHF* ultra-hypofractionation

^a^Equals power consumption multiplied by the emission factor of 0.38 $\frac{\mathrm{kg}\mathrm{C}\mathrm{O}_{2}}{\mathrm{kWh}}$ [25]

## Discussion

In this study, we carried out a quantitative analysis of treatment-related CO_2_ emissions to show the concrete saving potential of switching to moderate or ultra-hypofractionation for common RT indications. Depending on the indication, the treatment-related emissions could be reduced by up to 75% upon switching from an NF to a UHF concept, so that only around a quarter of the CO_2_ emissions would be generated. Previous publications on the subject have taken rough average values for the power consumption of linacs as the basis for calculation, whereas we have surveyed the actual power consumption of real treatment plans using current evidence-based concepts and treatment techniques [6, 12, 25]. The collected data form the basis of realistic calculations of electricity consumption, which has a significant impact on the carbon footprint. Assuming that patients do not suffer any medical disadvantage by switching to shorter—namely UHF—concepts, the electricity consumption and environmental impact of direct and indirect CO_2_ emissions should be relevant factors in medical decision-making. There is also a potentially significant improvement in patients’ quality of life, as the reduced number of irradiations leads to reduced travel times and less time spent at the hospital.

It becomes evident that the majority of the energy consumption and CO_2_ footprint of the linac for patient RT is not due to patient irradiation itself. In our patient groups, we consistently see the highest CO_2_ emissions due to patient travel, regardless of the fractionation concept and indication. In the breast, prostate, and bone cohorts, we see a travel-related CO_2e_ in the range of 67–82% (median 75%). Naturally, the shorter the concepts, the higher the proportion of travel-related CO_2e_. This corresponds quite closely to the data collected by Chuter et al. from the UK, where patient travel accounted for 76–86% of the total carbon footprint [6]. Bedir et al. recently presented a German analysis that quantitatively investigated the influence of patient travel in breast cancer patients as a function of fractionation [25]. The authors analyzed travel distances for more than 4000 breast cancer patients and showed that moderate hypofractionation can compensate up to 23%, and even more in the case of UHF, in comparison to NF.

Patient travel has a substantial impact on the environmental footprint of RT. It is therefore concluded that HF protocols can achieve a CO_2_ reduction of 77% [7]. The Federal Environment Agency in Germany states that approximately 0.22 t of carbon dioxide equivalents are produced for 1000 kWh of electricity [17]. With a hypothetical number of 100 breast cancer patients per year, switching from NF to UHF could save up to 2.7 t of CO_2e_ in a worst-case approximation if every patient travels by car. In the same way, switching bone metastasis RT from standard protocols to UHF for a total of 100 patients would save up to 1 t of CO_2e_ per year. In addition, the reduced patient traffic results in even higher values regarding the CO_2e_ reduction.

Our results support these statements. In conclusion, energy consumption is most constant and lowest with UHF, while it is highest and most variable in NF. This could mean that shorter treatment regimens (UHF) have not only temporal but also energetic advantages. Ultra-hypofractionated protocols reduced the energy consumption per treatment course for all entities compared to NF as well as HF. In relative terms, a reduced energy consumption of up to 5.5 times was achievable using UHF concepts. For the bone metastasis group, an increase in the interquartile range is observed in comparison to the other two entities. Reasons for this are the heterogeneous target volumes (only one vertebral body versus several vertebral bodies) and larger differences in the VMAT treatment plan (one rotation field versus several rotation fields). The RT technique was significantly more uniform in the prostate and breast collectives.

We were also able to prove the importance of the stand-by mode of the linear accelerator for saving energy. It is to be expected that the generation and acceleration of electrons and their conversion into X‑rays will require significant energy input. High electrical voltages in the megavolt range are required to accelerate the electrons. In order to accelerate the electrons sufficiently, microwaves are generated, which are also produced at high energy via magnetron tubes. In addition to pure beam generation, there are many processes that have to run continuously, such as those cooling the system and maintaining the vacuum as well as the basic control systems. Consideration should be given to ensure that the manual timers for waking up the linear accelerator are not set too generously for energy-smarter radiotherapy.

The energy consumption of our linear accelerator is substantially reduced in sleep mode (approximately 0.2 kWh). As soon as the linac is switched on and is in stand-by mode, the energy consumption rises sharply without the beam being on (3–4 kWh). This reinforces the importance of linear accelerator manufacturers offering a sleep mode. As soon as the beam is switched on, we detect peaks of up to 20 kWh, while these peaks are only very short lived. On average, the hourly energy consumption observed in this work was nearly 5 kWh in clinical applications. This shows that the main energy consumer is the maintenance of the preparatory status of the linac, which is consistent with results in the literature [12]. As a result, the hotspot of the carbon footprint throughout a linac lifecycle is its electricity consumption when in use. As a consequence, we changed the morning automatic start-up of the linac from 6 am to 6.45 am, so that the timers for magnetron and thyratron expire around 7 am. As a result, we save round about 15 kWh of energy per week without any restrictions for our department and regarding patient treatment time.

While our analysis was focused on the main factors influencing CO_2_ emissions in the radiotherapeutic pathway, other parameters have been ignored or simplified. For patient travel, we took an averaged distance of all patients based on a survey for one clinical workday in our department (57.7 km). However, this can vary greatly depending on rural or urban regions. We also made the simplified assumption that all patients travel by petrol car. We ignored the influence of SF_6_ gas (sulfur hexafluoride) leakage after talking to the manufacturer, who assured us that there is hardly any measurable leakage with this type of linac. The analysis also did not include other life cycle assessment-relevant items such as personal protective equipment, travel of the departmental staff, (cone-beam) CT-related power consumption, and other pretreatment imaging.

The generalizability of the results is limited by the rather small patient cohort (30 patients), the focus on only three specific entities (prostate cancer, breast cancer, and bone metastases), and the use of only one linear accelerator manufacturer and treatment planning system. Nevertheless, our findings show strong agreement with the current literature [7, 25]. The most significant impact on CO_2_ emissions comes from patient transport, which is directly related to fractionation. Potential for CO_2_ reduction exists in both fractionation protocols and linac operation (sleep mode) and should be fully exploited. This work aims to contribute to the emerging field of the CO_2_ impact of one of the most important cancer therapies, radiotherapy, by providing empirical evidence. It aims to raise awareness of the issue within the community and to encourage future efforts to consider environmental and resource conservation options. Future research should explore other tumor types to enhance the generalizability of findings, alongside the collection of long-term sustainability data. Investigating the integration of renewable energy into radiotherapy facilities and conducting life cycle analyses of radiotherapy equipment could further provide information regarding potential opportunities to reduce environmental impact and improve sustainability.

## Conclusion

Efficient energy management in radiation therapy is crucial for both environmental and economic sustainability while maintaining treatment quality. Our analysis shows that ultra-hypofractionated regimens offer significant energy savings by reducing the overall consumption. Technological advancements, such as modern accelerators with optimized radiation modes and power management, can further reduce the energy demand. A 75% reduction in CO_2_ emissions can be achieved through these treatment concepts, mainly by decreasing patient logistics. We demonstrated in our work that a climate-smart approach to cancer care is possible due to shorter treatment concepts. The use of UHF concepts is therefore desirable not only from a medical and patient care perspective, but also for climate-smart reasons.
